# Lymphocyte subsets for predicting inflammatory bowel disease progression and treatment response: a systematic review

**DOI:** 10.3389/fimmu.2024.1403420

**Published:** 2024-08-20

**Authors:** Rirong Chen, Chao Li, Jieqi Zheng, Zinan Fan, Li Li, Minhu Chen, Baili Chen, Shenghong Zhang

**Affiliations:** ^1^ Department of Gastroenterology, The First Affiliated Hospital, Sun Yat-sen University, Guangzhou, China; ^2^ Department of Clinical Medicine, Zhongshan School of Medicine, Sun Yat-Sen University, Guangzhou, China; ^3^ Guangxi Hospital Division of The First Affiliated Hospital, Sun Yat-sen University, Nanning, China

**Keywords:** inflammatory bowel disease, Crohn’s disease, ulcerative colitis, lymphocytes, disease progression, biologics response

## Abstract

**Background:**

Lymphocytes play a key role in the pathogenesis of inflammatory bowel disease (IBD) and are widely explored as promising prognostic indicators. We aimed to outline the existing evidences on the capability of lymphocyte subpopulations to predict disease progression and treatment response in patients with IBD.

**Methods:**

The protocol for this review was registered in PROSPERO (registration ID: CRD 42022364126). Systematic retrieval was conducted using PubMed, Embase, and Web of Science databases. Original articles on the prognostic value of lymphocyte subsets in IBD published up to April 8, 2023 were eligible for inclusion. The Newcastle–Ottawa Scale was used to evaluate the risk of bias.

**Results:**

Twenty studies were ultimately included: eight evaluated the prediction of disease progression and 12 focused on the prediction of treatment response. According to the Newcastle–Ottawa Scale, three studies were of high quality, 16 were of moderate quality, and only one was of low quality. T-cell subpopulations, including CD4^+^ T cells, CD8^+^ T cells, and γδ T cells, are revealed to have prognostic capacity. Transmembrane tumor necrosis factor α-bearing lymphocytes, CD4^+^ T cells, CD8^+^ T cells, and Plasma cells are found to have the potential to predict the response to anti-TNFα agents. In contrast memory T cells, CD4^+^ T cells, and naïve B cells may predict the response to vedolizumab.

**Conclusions:**

This systematic review identified several potential lymphocyte subset-related predictors. If verified in large cohort prospective studies, these findings could aid clinical decision-making.

**Systematic Review Registration:**

https://www.crd.york.ac.uk/PROSPERO/, identifier CRD42022364126.

## 1 Introduction

Inflammatory bowel disease (IBD), a chronic recurrent disorder of the gastrointestinal tract that results in abdominal discomfort, diarrhea, weight loss, and bloody stools, encompasses two main conditions: ulcerative colitis (UC) and Crohn’s disease (CD) (1). In UC, the lesions are limited to the colon and rectum, with superficial mucosal inflammation extending contiguously. UC can lead to fulminant colitis and toxic megacolon and in severe cases may require pancolectomy (2). In contrast, CD is characterized by relapsing transmural inflammation and involvement of the entire digestive tract, often in a fragmented manner. CD progression, involving the development of fistulas, abscesses, and strictures, can lead to disability and require surgery (3). The symptoms and progressive nature of IBD account for a poor quality of life for patients and an enormous burden on society. Moreover, IBD has become prevalent worldwide over the last decade, with a steadily increasing incidence (4). Therefore, the identification of disease prognostic factors that will aid in the selection of appropriate therapeutic strategies and allow personalized management of IBD is urgently required.

Although the pathogenesis of IBD is not fully understood, environmental, genetic, microbial, and immune factors have been shown to participate in its occurrence and development (5). The most widely accepted hypothesis is that IBD, which is characterized by chronic intestinal inflammation, results from a dysregulated immune response following an interplay between the host and gut microbiota, especially in genetically susceptible individuals (6). Therefore, many studies have been performed on immune imbalance, initially focusing on adaptive immunity and subsequently on innate immunity (5). Current evidence suggests that lymphocytes and their products are involved in mucosal immunity and exert a crucial role in the pathogenesis of IBD. These lymphocytes include B cells, helper T (Th) cells, regulatory T cells (Tregs), and memory T cells (7). For instance, the predominance of IgG over IgA has been confirmed in inflamed IBD tissue and may be a mechanism underlying IBD development (8, 9). Moreover, overactivation of Th1 and Th17 cells and functional deficiency of Tregs have been implicated in IBD (10–12). Thus, targeting Th1 and Th17 responses through lymphocyte trafficking or cytokine signaling is a major current therapeutic strategy for IBD (7).

In addition to exploring the role of lymphocyte subsets in the pathogenesis of IBD, much attention has been paid to their prognostic value. However, it remains to be determined whether lymphocyte subsets can predict disease progression and treatment response in IBD, and if so, which subsets are most useful. In this systematic review, we aim to summarize the potential value of different lymphocyte subsets for predicting disease progression and treatment response.

## 2 Methods

### 2.1 Data sources and searches

This systematic review was conducted in compliance with the PRISMA (Preferred Reporting Items for Systematic Reviews and Meta-Analyses) statement (13). The protocol was registered in PROSPERO (registration ID: CRD 42022364126). Systematic literature searches in PubMed, Embase, and Web of Science databases were performed to identify citations pertaining to the use of lymphocyte subsets in IBD prognosis up to April 8, 2023. “Inflammatory bowel diseases,” “lymphocyte subsets,” and “prognosis” were used as index terms or free-text words. Reference lists of the included articles were manually searched to identify additional relevant publications. The complete search strategies for all databases are provided in **Supplementary Table 1**.

### 2.2 Study selection

The inclusion criteria were as follows: (a) studies involving patients diagnosed with IBD, including CD, UC, and unclassified IBD; (b) studies relating to certain lymphocyte subtypes, including T cells, B cells, natural killer cells, and their subsets; and (c) prognostic studies with clearly predefined outcomes.

Studies were excluded if they were: (a) duplicates, (b) not original research, (c) not performed on humans, (d) only available as an abstract, or (e) in a language other than English. Considering the whole discussion in a recent meta-analysis (14), studies according to the predictive ability of basal plasmacytosis in relapse among patients with UC were dismissed.

Two reviewers (C. Li and R. Chen) independently screened the titles and abstracts of all the potentially relevant studies for appropriateness. Discordance in eligibility decisions was resolved by consensus.

### 2.3 Eligibility assessment and data extraction

The full texts of articles identified by the preliminary screening procedure were further evaluated and analyzed. Relevant information was extracted independently by two reviewers (C. Li and J. Zheng) using a predesigned data extraction form. In cases of disagreement between the two reviewers, papers were re-evaluated by a third author (R. Chen) to reach a consensus.

The following data were extracted from ultimately included studies: author names; publication year; country; study design; IBD phenotypes and sample size; definition of outcomes; medication during follow-up; lymphocyte subsets; and prediction performance, such as the area under the receiver operating characteristic curve (AUC), sensitivity, specificity, and hazard ratio (HR).

### 2.4 Study quality assessment

The Newcastle–Ottawa Scale (NOS) (15) is a “9-star system” constructed in which a non-randomized study is evaluated in three broad respects: the selection of participants; the comparability of the study groups; and the attainment of either exposure or outcome for case-control or cohort studies, respectively. Two independent reviewers (Z. Fan and C. Li) applied this scale to appraise the quality of the selected studies. Total NOS scores of 8–9, 5–7, and 0–4 reflected high-, moderate-, and low-quality studies, respectively. Any discrepancies were discussed, and the results were tabulated to reach a consensus of opinion.

## 3 Results

### 3.1 Study selection and characteristics

**Figure 1:**
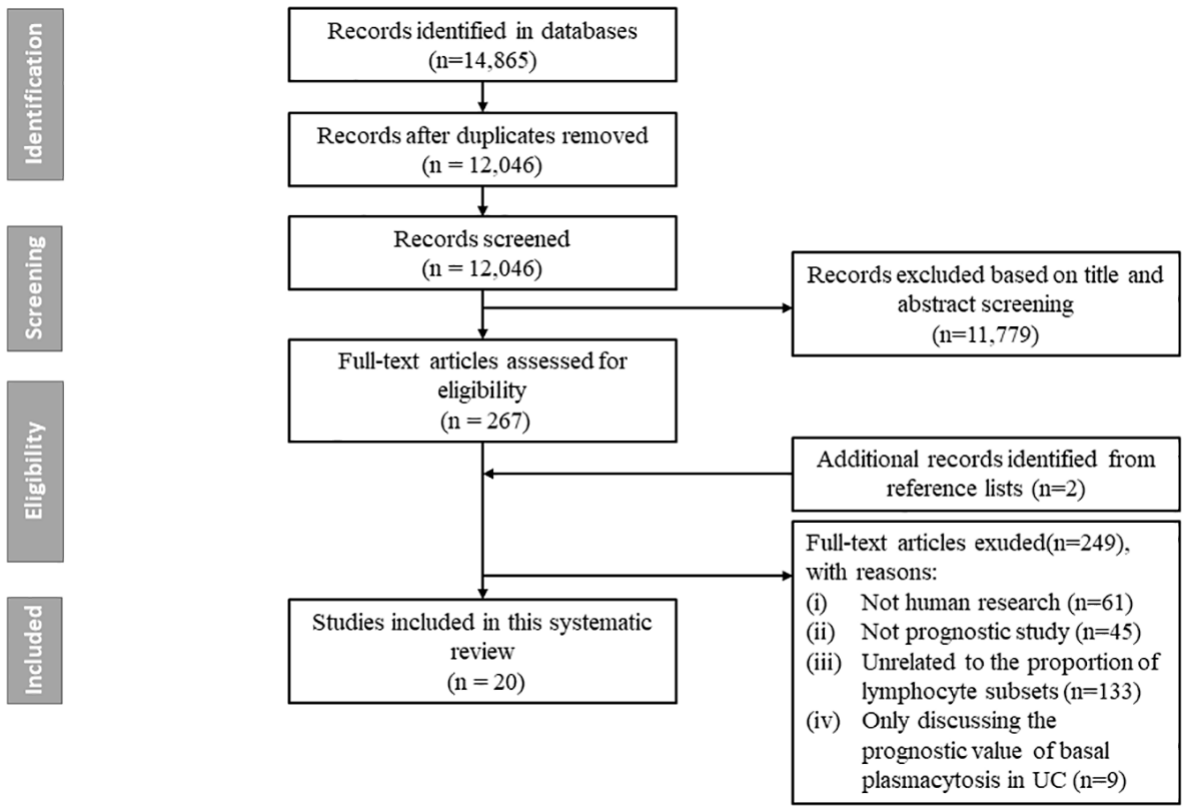
Flow chart of study selection. The literature search generated 14, 865 citations: 3, 241 in Medline (PubMed), 5, 481 in Embase (Ovid), and 6, 143 in Web of Science (Core Collection). After removing duplicates, 12, 046 studies remained. Next, 11, 779 citations were excluded when screening titles and abstracts; the full text and reference lists of the remaining 267 articles were reviewed. Two additional studies were identified from the reference lists, and in total 20 studies were finally included in the systematic review.

**Figure 2:**
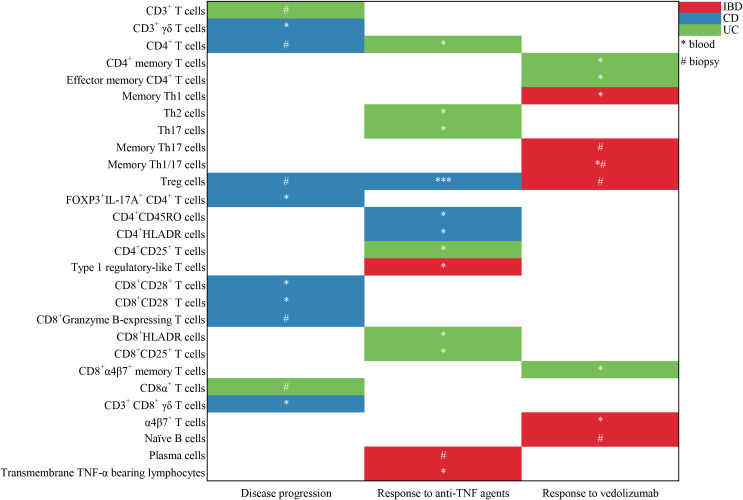
Heatmap to summary the included literatures about the predictive value of lymphocyte subsets in the prediction of disease progression and response to anti-TNFα agents and vedolizumab. * represents the lymphocyte subgroups from the peripheral blood; # represents the lymphocyte subpopulations from the mucosal biopsy. One symbol (* or #) means there is one corresponding study.

The literature search generated 14,865 citations: 3,241 in Medline (PubMed), 5,481 in Embase (Ovid), and 6,143 in Web of Science (Core Collection) (**Figure 1**; **Supplementary Table 1**). After removing duplicates, 12,046 studies remained. Next, 11,779 citations were excluded when screening titles and abstracts; the full text and reference lists of the remaining 267 articles were reviewed. Two additional studies were identified from the reference lists, and in total 20 studies were included in the systematic review (16–35). Among these studies, nine assessed the predictive value of lymphocytes in IBD, eight in CD, and three in UC (**Table 1**). Based on the outcome, these studies could be divided into two groups: disease prognosis (n = 8) and therapy response (n = 12). Eighteen studies recruited patients prospectively, one was retrospective cohort studies, and one was a bioinformatics analysis using data from the Gene Expression Omnibus (GEO) and Sequence Read Archive (SRA) databases. A heatmap was plotted to comprehensively summarize the included literatures (**Figure 2**).

**Table 1:** Characteristics of the included studies.

Author	Publication Year	Country	Study Design	Participants	Age, years	Male (%)	Follow-up time
Allez et al. (30)	2019	France	Prospective cohort	57 CD	Mean: 37.4 (range: 20–70)	32 (56)	Median: 6.9 months
Amini et al. (23)	2017	Iran	Prospective cohort	34 CD, 13 UC	CD: 38.7 ± 14.6; UC: 38.7 ± 14.6	UC: 7 (53.8); CD: 18 (52.9)	14 weeks
Andreu-Ballester et al. (26)	2020	Spain	Prospective cohort	102 CD	39.1 ± 13.9	57 (55.9)	–
Boschetti et al. (25)	2016	France	Prospective cohort	25 CD	–	–	12 months
Chao et al. (27)	2014	China	Prospective cohort	46 CD	Mean: 28 (range: 11–59)	29 (63.0)	Median: 30 (range: 26–37) months
Coletta et al. (34)	2020	Italy	Prospective cohort	18 CD, 20 UC	CD: 42 ± 13; UC: 45 ± 15	CD: 14 (77.8); UC: 12 (60.0)	14 weeks
Dai et al. (24)	2017	China	Retrospective cohort	65 CD	Complicated CD: 24.55 ± 5.79; control CD: 28.56 ± 7.13	46 (70.8)	30 weeks
Di Sabatino et al. (20)	2010	Italy	Prospective cohort	20 CD	Mean: 32.7 (range: 20–64)	11 (55)	10 weeks
Dige et al. (16)	2011	Denmark	Prospective cohort	26 CD	–	14 (53.8)	26 weeks
Duclaux-Loras et al. (29)	2022	France	Prospective cohort	113 CD	Mean: 38.3 (range: 18.1-79.1)	44 (38.9)	1 year
Dulic et al. (22)	2020	Hungary	Prospective cohort	16 CD, 16 UC	CD: mean, 30 (range: 18–48); UC: 42 (20–65)	CD: 9 (56.3); UC: 9 (56.3)	CD: 35 (16–47) months; UC: 24 (2–46) months
Gaujoux et al. (18)	2019	Israel	Prospective cohort	72 IBD	–	–	14 weeks
Gonzalez-Vivo et al. (35)	2022	Spain	Prospective cohort	15 UC	Remitters: median, 45 (IQR: 31.5–66); nonremitters: 41.5 (33.5–52.8)	8 (53.3)	14 weeks
Kotsafti et al. (31)	2019	Hungary	Prospective cohort	90 UC	–	–	Median: 17 (IQR: 12–25) months
Li et al. (21)	2015	Belgium	Prospective cohort	40 IBD	–	–	14 to 22 weeks
Magnusson et al. (17)	2013	Sweden	Prospective cohort	32 UC	Responders: median, 33 (IQR: 27–40); non-responder: 35 (23–46)	22 (68.8)	3 - 4 months
Shi et al. (19)	2021	China	Prospective cohort	139 IBD	–	–	4–6 weeks
Smids et al. (28)	2018	Netherlands	Prospective cohort	77 CD, 33 UC	–	–	> 1 year
Ungar et al. (32)	2018	Israel	Prospective cohort	39 IBD	–	–	14 weeks
Verstockt et al. (33)	2020	Belgium	Prospective cohort	11 CD, 20 UC	Median: 45.3 (IQR: 29.6–56.3)	14 (45.2)	CD: 6 months; UC: 14 weeks

IBD, inflammatory bowel disease; CD, Crohn’s disease; IQR, interquartile range; UC, ulcerative colitis.

### 3.2 Study quality assessment

The results of the NOS assessment of eligible studies are presented in **Supplementary Table 2**. Of the 20 studies, 3 (15%) were considered to be of high quality, 16 (80%) were of moderate quality, and only 1 (5%) was of low quality.

### 3.3 Lymphocyte subsets and disease progression

Eight studies (24–31) evaluated the prognostic value of specific lymphocyte subsets in either disease progression or postoperative recurrence; all focused on T cells (**Table 2**).

**Table 2:** Lymphocyte subsets for predicting disease progression in inflammatory bowel disease.

Lymphocyte subsets	Samples	Participants	Outcomes	Prognostic value	References
CD3^+^ CD8^+^ γδ T cells	Blood	102 CD	Surgery	Adjusted HR: 3.053 [95%CI, 1.098-8.488; P = 0.032])	Andreu-Ballester et al. (26)
CD3^+^ γδ T cells	Blood	102 CD	Surgery	Adjusted HR: 3.112 [95%CI, 1.140-8.496; P = 0.027])	Andreu-Ballester et al. (26)
Treg/Th1 ratio	Blood	46 CD	Endoscopic or clinical relapse	Compared to patients with recurrence, patients maintaining remission had a higher ratio (0.6 ± 0.2 vs 0.3 ± 0.1, P = 0.042)	Chao et al. (27)
Treg/Th17 ratio	Blood	46 CD	Endoscopic or clinical relapse	Compared to patients with recurrence, patients maintaining remission had a higher ratio (3.1 ± 1.1 vs 1.7 ± 1.2, P = 0.031)	Chao et al. (27)
CD8^+^CD28^+^ T cells	Blood	65 CD	Progression into active stage	AUC: 0.802 (95%CI: 0.697–0.907; P < 0.0001)	Dai et al. (24)
CD8^+^CD28^–^ T cells	Blood	65 CD	Progression into active stage	AUC: 0.338 (95%CI: 0.206–0.470; P = 0.042)	Dai et al. (24)
CD8^+^CD28^+^/CD8^+^CD28^–^ ratio	Blood	65 CD	Progression into active stage	AUC: 0.890 (95%CI: 0.822–0.958; P < 0.0001)	Dai et al. (24)
FOXP3^+^IL-17A^+^ CD4^+^ T cells	Blood	113 CD	Clinical relapse	Adjusted OR: 2.81 (95%CI: 1.13–7.04, P = 0.03)	Duclaux-Loras et al. (29)
CD3^+^ cells	Biopsy	33 UC	Treatment escalation	High percentages of CD3^+^ cells was associated with the treatment escalation (median: 62% [50–71] vs. 48% [IQR, 43–63], P = 0.03)	Smids et al. (28)
CD4^+^ cells	Biopsy	77 CD	Develop to stricturing or penetrating disease [B2/B3]	Higher percentage of CD4^+^ cells was associated with developing to B2/B3 disease (median: 74% [IQR: 65–79] vs. 65% [52–73], P = 0.02)	Smids et al. (28)
Treg cells	Biopsy	77 CD	Develop to stricturing or penetrating disease [B2/B3]	Higher percentage of Treg cells was associated with developing to B2/B3 disease (median: 15% [IQR 9–23] vs.11% [IQR 7–15], P = 0.04)	Smids et al. (28)
Treg cells	Biopsy	77 CD	Surgery	Higher percentage of Treg cells was associated with need for surgery (median: 15% [IQR 14–19] vs. 10% [7–15], P = 0.014)	Smids et al. (28)
Treg cells	Biopsy	77 CD	Treatment escalation	Higher percentage of Treg cells was associated with treatment escalation (median: 11% [8–16] vs. 8% [IQR 5–10], P = 0.014)	Smids et al. (28)
CD8α^+^ T cells	Biopsy	90 UC	Low-grade neoplasia	AUC: 0.74 (95% CI: 0.62–0.84, P = 0.004)	Kotsafti et al. (31)
Granzyme B-expressing CD8^+^ T cells	Biopsy	25 CD	Postoperative recurrence	The frequency was higher in patients with recurrence at 12 months than those who remained in remission (10.24 ± 4.67% vs. 5.98 ± 3.28%; P < 0.05)	Boschetti et al. (25)
T cell clonal expansions	Biopsy	57 CD	Postoperative recurrence	AUC: 0.69 (95%CI: 0.54-0.83)	Allez et al. (30)

IBD, inflammatory bowel disease; CD, Crohn’s disease; UC, ulcerative colitis; IQR, interquartile range; Treg cell, regulatory T cell; Foxp3, Forkhead box protein P3; IL, interleukin.

Four studies (24, 26, 27, 29) reported the prognostic performance of circulating lymphocytes. A retrospective cohort study by Dai et al. (24) assessed the ability of the CD8^+^CD28^+^/CD8^+^CD28^–^ cell ratio to predict disease progression in 65 newly diagnosed patients with CD. Baseline CD8^+^CD28^+^ and CD8^+^CD28^–^ T cell percentages, and their ratio, were shown to have predictive value, with AUCs of 0.802, 0.338, and 0.890, respectively. A prospective cohort study that enrolled 102 patients with CD reported that lower levels of circulating CD3^+^ γδ and CD3^+^CD8^+^ γδ T cells were associated with a higher risk of surgery, with adjusted HRs of 3.112 (95% confidence interval [CI], 1.140–8.496) and 3.053 (95%CI, 1.098–8.488), respectively (25). The two additional studies reported the prognostic value of CD4^+^ T cell subgroups. Chao et al. (27) plotted the balance between Tregs, Th1 cells, and Th17 cells in 46 patients with CD. Although significant differences were not observed between the cell subtypes, further analysis identified the baseline Treg/Th1 and Treg/Th17 ratios as prognostic indicators for endoscopic or clinical relapse. Duclaux-Loras et al. (29) prospectively analyzed the association between circulating CD4^+^ T-cell subpopulations and the risk of CD relapse. The absolute number of FOXP3^+^IL-17A^+^CD4^+^ T cells was detected to be an independent predictor of further clinical relapse, when measured within the four months prior to relapse, with an adjusted odds ratio of 2.81 (95%CI: 1.13–7.04) when transformed into a binary variable with a cut-off value of 1.4 cells/mm^3^.

Additional four studies analyzed lymphocyte subsets in biopsies (25, 28, 30, 31). A prospective cohort study of patients with CD (30) uncovered a moderate ability of T cell clonal expansion to predict early postoperative endoscopic recurrence, with an AUC of 0.69 (95%CI: 0.54–0.83). Further analyses indicated that clonal expansion associated with postoperative recurrence primarily occurred within the CD8^+^ T-cell compartment. Meanwhile, Boschetti et al. (25) depicted a significant increase in granzyme B-expressing CD8^+^ T cells in the ileal lamina propria 6 months after ileocolonic resection in patients who were in endoscopic remission at 6 months but developed further endoscopic recurrence at 12 months compared with that in those who sustained remission for one year. Kotsafti et al. (31) conducted a prospective cohort study to identify immunological markers for the presence and persistence of low-grade neoplasia in patients with UC. Baseline CD8α^+^ T cells infiltration in the lamina propria was greater in patients who were subsequently diagnosed with low-grade neoplasia at the second colonoscopy than in those who were not and had a moderate predictive performance, with an AUC of 0.74 (95%CI: 0.62–0.84); however, this predictive value was lost following adjustment for patient age. Smids et al. (28) carried out a prospective cohort study involving newly diagnosed patients with IBD to systematically investigate the association between T subsets at first presentation and variable disease outcomes. Among newly diagnosed patients with CD, higher baseline percentages of CD4^+^ T cells and Tregs were associated with progression to structuring or penetrating disease. Baseline percentages of Tregs were also higher in newly diagnosed patients with CD who subsequently required abdominal surgery or more aggressive treatment, compared with those who did not. In patients with UC, a higher proportion of CD3^+^ T cells at baseline was associated with the need for treatment escalation with immunomodulators (28).

### 3.4 Lymphocyte subsets and treatment response

Twelve studies explored the ability of lymphocyte subpopulations to predict the response to treatment with biological therapeutics, including anti-tumor necrosis factor α (anti-TNF-α) agents (infliximab [IFX] and adalimumab [ADA]) and an anti-α4β7 integrin agent (vedolizumab).

#### 3.4.1 Lymphocyte subsets and response to anti-TNF-α agents

A total of eight studies (16–23) have analyzed the ability of a variety of lymphocyte subsets in peripheral blood or biopsy samples to predict non-response to anti-TNF-α therapy in patients with IBD (**Table 3**).

**Table 3:** Lymphocyte subsets for predicting therapy response to anti-tumor necrosis factor agents.

Lymphocyte subsets	Samples	Participants	Biologics	Definition of response	Predictive value	References
Transmembrane TNF-α bearing lymphocytes	Blood at baseline	34 CD, 13 UC	IFX	Endoscopic response	For mean fluorescence intensity, AUC: 0.827 (95% CI, 0.695–0.960; P<0.001)	Amini et al. (23)
CD4+CD45RO cells	Blood at baseline	16 CD	IFX/ADA	Clinical response	AUC: 1.0 (P = 0.03)	Dulic et al. (22)
CD4+HLADR cells	Blood at baseline	16 CD	IFX/ADA	Clinical response	Pearson’s r: 0.50 (P = 0.058)	Dulic et al. (22)
CD4^+^ cells	Blood at baseline	16 UC	IFX/ADA	Clinical response	AUC: 0.85 (P= 0.08)	Dulic et al. (22)
Th2 cells	Blood at baseline	16 UC	IFX/ADA	Clinical response	Pearson’s r: 0.59 (P = 0.03)	Dulic et al. (22)
Th17 cells	Blood at baseline	16 UC	IFX/ADA	Clinical response	Pearson’s r: 0.61 (P = 0.03)	Dulic et al. (22)
CD8+HLADR cells	Blood at baseline	16 UC	IFX/ADA	Clinical response	Pearson’s r: 0.59 (P = 0.06)	Dulic et al. (22)
Treg cells	Blood at baseline	20 CD	IFX	Clinical response	The mean percentage of Treg was lower in responders than non-responders (2.6% ± 1.1 vs. 7.3% ± 1.8; P < 0.0001)	Di Sabatino et al. (20)
Treg cells	Blood at baseline	26 CD	ADA	Clinical response	The mean percentage of Treg was higher in responders than non-responders (4.0% [IQR 3.9–4.1%] vs. 5.1% [IQR 4.6–6.4%]; P =0.005)	Dige et al. (16)
Treg cells	Blood at baseline	40 IBD	IFX	Clinical response	AUC: 0.82 (P = 0.0001)	Li et al. (21)
CD45RA^+^Foxp3^lo^ resting Treg cells	Blood at baseline	40 IBD	IFX	Clinical response	AUC: 0.81 (P = 0.001)	Li et al. (21)
CD45RA^-^Foxp3^hi^-activated Treg cells	Blood at baseline	40 IBD	IFX	Clinical response	AUC:0.74 (P = 0.015)	Li et al. (21)
Type 1 regulatory-like T cells	Blood at baseline	40 IBD	IFX	Clinical response	AUC: 0.73 (P = 0.02)	Li et al. (21)
CD25^+^CD4^+^ T cells	Blood at week 2 and baseline	34 UC	IFX	Clinical response	AUC: 0.92	Magnusson et al. (17)
CD25^+^CD8^+^ T cells	Blood at week 2 and baseline	34 UC	IFX	Clinical response	AUC: 0.91	Magnusson et al. (17)
Plasma cells	Biopsy at baseline	72 IBD	IFX	Clinical and/or endoscopic improvement	Exploring cohort: AUC=0.81 External validation cohort: AUC=0.74	Gaujoux et al. (18)
GIMATS (IgG plasma cells, inflammatory mononuclear phagocytes, activated T cells, and stromal cells)	Biopsy at baseline	139 IBD	IFX/ADA	Endoscopic response	Microarray dataset: AUC = 0.853 (95%CI: 0.778-0.928) RNA-seq dataset: AUC = 0.720 (95%CI: 0.559-0.881)	Shi et al. (19)

IBD, inflammatory bowel disease; CD, Crohn’s disease; UC, ulcerative colitis; IQR, interquartile range; Treg cell, regulatory T cell; HLADR, human leukocyte antigen DR; IFX, infliximab; ADA, adalimumab.

Six of these studies (16, 17, 20–23) evaluated lymphocyte subsets in peripheral blood. Amini Kadijani et al. (23) observed a borderline significant difference in baseline transmembrane TNF-α-bearing lymphocytes between responders and non-responders. Furthermore, a higher baseline mean fluorescence intensity of transmembrane TNF-α was observed in IFX responders than in non-responders. The AUC was 0.827 (*P* = 0.001), with a sensitivity of 83.3% and a specificity of 88.2%, using the optimal cutoff value of 30.5. Studies on T cells and their subsets have also been performed. Dulic et al. (22) carried out a prospective study measuring the prevalence of 14 T cell subsets in peripheral blood. Analyses showed that a percentage of CD4^+^CD45RO T cells lower than 49.05% at baseline predicted treatment response in patients with CD with a sensitivity of 100% and a specificity of 92.3% (AUC, 1.0; *P* = 0.03), whereas Th2 and Th17 cell frequencies were positively correlated with response duration in patients with UC, with Pearson correlation coefficients of 0.59 and 0.61, respectively. Three studies focused on total circulating Tregs (16, 20, 21). The earliest study showed that baseline Treg frequency was significantly higher in non-responders than in responders in a small cohort of patients with CD (20). However, results from the two subsequent studies contradicted these initial findings (16, 21). Both reported that a higher baseline percentage of Tregs as a proportion of the CD4^+^ T cell population was associated with a greater likelihood of remission in patients with CD and IBD. Meanwhile, a study in an independent UC cohort showed no baseline difference in Treg proportion with regard to the IFX treatment response measured between the third and fourth treatment dose (17). Furthermore, Li et al. (21) revealed that the percentages of resting Tregs, activated Tregs, total Tregs, and type-1 regulatory like T cells among CD4^+^ cells all performed well in the prediction of the clinical response to IFX 14–22 weeks after the start of therapy in patients with IBD. Magnusson et al. (17) attempted to identify predictive biomarkers by examining differences between baseline and two weeks after the initial infusion after failing to observe any baseline differences in T cell subsets between responders and non-responders. The results indicated that reduced frequencies of CD25^+^CD4^+^ and CD25^+^CD8^+^ T cells at two weeks after the first infusion could discriminate between responders and non-responders, with AUCs of 0.92 and 0.91, respectively.

Two studies (18, 19) analyzed lymphocyte frequency in biopsy samples rather than peripheral blood. Gaujoux et al. (18) conducted a prospective cohort study and determined that a high proportion of plasma cells in biopsies is a robust baseline predictor of non-response to anti-TNF-α agents in patients with IBD. These findings were subsequently verified in two independent real-life cohorts; plasma cell proportion had the greatest predictive value following subgroup analysis that included only highly inflamed tissues, with an AUC of 0.82. Moreover, a bioinformatics study (19) based on GEO and SRA databases constructed a pathogenic cellular module termed GIMATS, which involved the concentration of IgG-producing plasma cells, inflammatory monocytes, activated T cells, and stromal cells in biopsy samples from patients with IBD (8). Analyses in microarray and RNA sequencing datasets established that the GIMATS module had a satisfactory overall performance for the prediction of non-response to anti-TNF-α agents, irrespective of laboratory techniques. Patients with high GIMATS module scores had a significantly worse response to anti-TNF-α therapy than those with low GIMATS module scores.

#### 3.4.2 Lymphocyte subsets and response to anti-α4β7 integrin agent

Five studies (19, 32–35) explored the ability of lymphocyte subsets to predict treatment responses to the anti-α4β7 integrin agent, vedolizumab (**Table 4**). Two collected samples from peripheral blood (32, 35), two were from biopsy (19, 33), and one was from both (34).

**Table 4:** Lymphocyte subsets for predicting therapy response to vedolizumab.

Lymphocyte subsets	Samples	Participants	Definition of response	Predictive value	References
CD4^+^ memory T cells	Blood at baseline	15 UC	Clinical remission	Patients who achieved remission presented higher concentration than those who were did not (median: 394.47 vs. 304.73 cells/ml, P = 0.02).	Gonzalez-Vivo et al. (35)
CD4^+^ memory T cells	Blood at baseline	15 UC	Biochemical remission or endoscopic improvement	Patients who were in remission had higher concentration than those were not (median: 394.47 vs. 304.73 cells/ml, P = 0.02).	Gonzalez-Vivo et al. (35)
CD4^+^ memory T cells	Blood at baseline	15 UC	Sustained clinical remission	Patients who were in remission presented higher concentration compared with non-remitters (median: 394.47 vs. 327.66 cells/ml, P = 0.02).	Gonzalez-Vivo et al. (35)
Memory Th1 cells	Blood at baseline	18 CD, 20 UC	Short-term clinical remission	Higher levels were associated with clinical remission both in the total IBD cohort (18.3% in remitters vs 12.3% in non-remitters, P = 0.02) and when CD and UC patients were separately analyzed (P <0.05).	Coletta et al. (34)
Memory Th1 cells	Blood at baseline	18 CD, 20 UC	Long-term clinical remission	Higher levels were significantly associated with clinical remission (*P* = 0.008).	Coletta et al. (34)
Memory Th1 cells	Blood at week 14 and baseline	18 CD, 20 UC	Long-term clinical remission	Patients not achieving Week 54 clinical remission were characterized by a significantly higher increase compared with remitters (*P* < 0.01).	Coletta et al. (34)
Memory Th1/17 cells	Blood at baseline	18 CD, 20 UC	Short-term clinical remission	Higher levels were associated with clinical remission both in the total IBD cohort [3.8% vs 1.0%, P = 0.012] and in CD patients [5.2% vs 1.5%, P = 0.026], but not in UC patients (P > 0.05).	Coletta et al. (34)
CD8^+^ α4β7^+^ memory T cells	Blood at baseline	15 UC	Clinical remission	Patients who achieved remission presented higher concentration than those who did not (19.27 vs. 11.63 cells/ml, P = 0.02).	Gonzalez-Vivo et al. (35)
CD8^+^ α4β7^+^ memory T cells	Blood at baseline	15 UC	Biochemical remission or endoscopic improvement	Patients who were in remission had higher concentration than those were not (median: 14.43 vs. 11.63 cells/ml, P = 0.02).	Gonzalez-Vivo et al. (35)
CD8^+^ α4β7^+^ memory T cells	Blood at baseline	15 UC	Sustained clinical remission	Patients who were in remission presented higher concentration compared with non-remitters (median: 14.43 vs. 11.85 cells/ml, P = 0.02).	Gonzalez-Vivo et al. (35)
α4β7^+^ T cells	Blood at baseline	39 IBD	Clinical response	Cannot predict	Ungar et al. (32)
Memory Th17 cells	Biopsy at baseline	18 CD, 20 UC	Endoscopic response	Reduced proportions were associated with endoscopic response in the total IBD cohort (P = 0.012) and in UC patients.	Coletta et al. (34)
Memory Th17 cells	Biopsy at baseline	18 CD, 20 UC	Long-term clinical remission	Lower levels were significantly associated with clinical remission (*P* = 0.035).	Coletta et al. (34)
Memory Th1/17 cells	Biopsy at baseline	18 CD, 20 UC	Endoscopic response	Reduced proportions were associated with endoscopic response in the total IBD cohort (*P* = 0.005) and in UC patients.	Coletta et al. (34)
Memory Th1/17 cells	Biopsy at baseline	18 CD, 20 UC	Long-term clinical remission	Lower levels were significantly associated with clinical remission at Week 54 (*P* = 0.018).	Coletta et al. (34)
Effector memory CD4^+^ T cells	Biopsy at baseline	11 CD, 20 UC	Endoscopic remission	A significant enrichment in non-remitters (P = 0.008).	Verstockt et al. (33)
Treg cells	Biopsy at baseline	11 CD, 20 UC	Endoscopic remission	A significant enrichment in non-remitters (*P* = 0.05).	Verstockt et al. (33)
Naïve B cells	Biopsy at baseline	11 CD, 20 UC	Endoscopic remission	A significant enrichment in remitters (P = 0.03).	Verstockt et al. (33)
GIMATS (IgG plasma cells, inflammatory mononuclear phagocytes, activated T cells, and stromal cells)	Biopsy at baseline	84 IBD	Endoscopic response	Microarray dataset: AUC:0.661 (95% CI, 0.395-0.927) RNA-seq dataset: AUC =0.728 (0.583-0.873).	Shi et al. (19)

IBD, inflammatory bowel disease; CD, Crohn’s disease; UC, ulcerative colitis; IQR, interquartile range; Treg cell, regulatory T cell.

Gonzalez-Vivo et al. (35) assessed the ability of baseline peripheral blood CD4^+^ and CD8^+^ memory T-cells to predict future response to vedolizumab treatment in patients with UC and reported a robust correlation between higher concentrations of CD4^+^ memory T cells and CD8^+^α4β7^+^ memory T cells and short-term (14 weeks) biochemical, endoscopic, and clinical improvement as well as sustained (52 weeks) clinical remission. However, a study comprising a prospective cohort (n=13) and an additional exploratory cohort (n=26) failed to confirm the ability of the baseline proportion of α4β7^+^ T cells to predict response to treatment (32).

Verstockt et al. (33) identified significant enrichment of effector memory CD4^+^ T cells (*P* = 0.008) and Tregs (*P* = 0.005) in biopsies from patients with IBD who failed to enter endoscopic remission at week 14, whereas naïve B cells were enriched in patients who did enter remission (*P* = 0.03). Shi et al. (19) applied their GIMATS module, which predicted anti-TNF-α treatment responses, to the prediction of treatment responses to vedolizumab. A reliable predictive capacity was established, with AUCs of 0.853 (95%CI: 0.778–0.928) and 0.720 (95%CI: 0.559–0.881) for the microarray and RNA sequencing datasets. Contrary to the negative correlation between high GIMATS module scores and the anti-TNF-α treatment response, a positive association was observed between higher scores and improved clinical outcomes following vedolizumab treatment.

Coletta et al. (34) conducted a phase IV exploratory interventional trial in which baseline peripheral blood and biopsy samples were both obtained. A higher baseline level of circulating Th1 memory cells was found to significantly correlate with the clinical response at week 14 in CD, UC and the entire cohort. In contrast, decreased levels of lamina propria Th17 and Th1/17 memory cells were observed to be related to endoscopic response in the total IBD cohort and in the sole UC cohort. The relevance of these findings re-emerged when concerning clinical remission at week 54. Among patients receiving vedolizumab treatment for one year, those who failed to achieve clinical remission at the endpoint exhibited growth in peripheral blood Th1 memory cells at week 14 compared to baseline levels.

## 4 Discussion

Lymphocyte subsets, which are demonstrated to exert a principal role in the pathogenesis of IBD, have attracted increasing attention in recent decades for their prognostic and predictive value (7). This review is the first to systematically outline the current evidence for the predictive value of lymphocyte subsets in IBD. We found a satisfactory capacity of lymphocyte subpopulations to predict disease prognosis and treatment response. The lymphocyte subsets analyzed varied from memory to effector lymphocytes, T cell subpopulations to plasma cells, and from single predictors to ratios and lymphocyte subset-based cellular modules. Although more high-quality studies are necessary for clinical application, this review highlights the potential of several lymphocyte subsets to predict disease progression and treatment response in patients with IBD. These findings may aid scientists and clinicians in the implementation of personalized management for patients with IBD.

IBD is characterized by a complicated and chronic disease course, and affects patients both physiologically and psychologically. In CD, whereas some patients experience relatively mild disease requiring only limited immunomodulation and with few severe complications, a considerable proportion progress to destructive penetrating and/or stricturing disease with variable complications, necessitating repeated biological therapy and surgery (36–38). Epidemiological studies have shown that CD carries a 10- and 20-year cumulative risk for surgical intervention of approximately 38% and 70%, respectively (39, 40). Nevertheless, surgical therapy rarely cures CD, and postoperative therapy is frequently challenging (41). A similarly diverse range of outcomes is observed in patients with UC; many require advanced treatment and surgery, while others require only oral or topical mesalazine treatment. Approximately 10% and 15% of patients with UC undergo subtotal colectomy within 5 and 10 years of diagnosis, respectively, and UC is reported to carry up to a 5% risk of developing colorectal cancer within 30 years (38, 42). Considering the complex course of IBD, the prediction of disease progression is desirable to select prophylaxis following consideration of the risk–benefit ratio, which would offer patients with a high likelihood of undergoing a complicated trajectory a tailored treatment plan and avoid unnecessarily exposing patients who are likely to experience a mild disease course to immune suppression or potential adverse effects.

Current evidence suggests that immunological factors play a central role in the pathogenesis of IBD, and other components, including environmental, genetic, and microbial factors, may trigger dysregulated immune responses that are responsible for the chronic intestinal inflammation typical of CD and UC (5). Many lymphocyte subpopulations have been linked to disease prognosis, including surgery, relapse, and postoperative recurrence. In this review, we revealed that various lymphocyte subsets have a promising ability to predict disease progression in patients with IBD. T-cell subpopulations, including CD4^+^ T cells, CD8^+^ T cells, and γδ T cells, are revealed to have prognostic capacity (24–31). These findings improve understanding of the pathogenesis of IBD, and aid in the development of personalized medicine, which requires accurate prediction.

As widely-studied biologics, anti-TNFα agents are found to act by blocking soluble and transmembrane TNF-α, inducing antibody- or complement-dependent cellular toxicity, and direct or indirect apoptosis of TNF-α–producing macrophages and T cells, and inducing the development of Treg cells and regulatory macrophages (43, 44). Besides, Vedolizumab is a humanized monoclonal antibody that works mainly through specific recognition and blockade of the α4β7 heterodimer, predominantly on memory T cells, and then selectively blocking gut lymphocyte trafficking (45, 46) while ustekinumab is a monoclonal antibody targeting against the p40 subunit of interleukin-12 and interleukin-23 (47). Despite the clinical and endoscopic efficacy and safety of biological therapeutics demonstrated in both clinical trials and real-world studies (48–51), a considerable percentage of individuals do not respond to these treatments, and are therefore unnecessarily, and at great cost, exposed to potential adverse events (52–54). Accumulating evidence suggests that responsiveness to biological therapy in patients with IBD is driven by multiple factors, including clinical disease phenotype, genetic factors, local microenvironment, microbiota, and pharmacological factors (55). Current knowledge suggests that immune cells, especially lymphocytes, play a crucial role in molecular resistance to treatment, being involved in interactions between the immune system and microbes, and immunogenetic and pharmacological mechanisms (e.g., anti-drug antibodies) (55–57). In several studies included in this review, the targeted lymphocyte subsets were explored to have the capacity of predicting response, such as Transmembrane TNFα bearing lymphocytes (23) for response to anti-TNFα drugs and CD8^+^α4β7^+^ memory T cells (35) for response to anti-α4β7 monoclonal antibody, vedolizumab. It can be reasonably explained by the mechanistic failure of specific biologics: a lower concentration of target lymphocyte subsets, which may be complicatedly determined by disease condition, genetic factors and unknown pathogenesis, correspond to a weaker function of blockade of the specific immune signaling pathway using selective antibodies (55). Moreover, a distinct level of various lymphocyte subpopulations, name d cellular heterogeneity, would indirectly reflect the baseline level of T cell-dominant pathogenesis response in mucosal lesions or link with the expression of target molecules (e.g., membrane-bound TNF-α), and hence correlate with the molecular resistance and mechanistic failure of biologics (8, 55). For instance, Martin et al. explored a GIMATS module, whose presence at the diagnosis is related to failure to achieve durable clinical remission upon anti-TNF therapy, by single-cell techniques to mucosal lesions from patients with ileal CD and identified a distinct network connectivity that are demonstrated to drive the GIMATS module (8).

Hence, accompanied by mechanistic research on non-response to biological therapeutics, efforts have been made to identify credible lymphocyte subset-related biomarkers to facilitate personalized management of IBD. Apart from cytokines that have been explored to have prognostic value in the prediction of biologics efficacy and summarized elsewhere (58–61), lymphocyte subsets from peripheral blood or biopsy samples have also been suggested to have predictive potential. Our review illustrates the specific role of certain lymphocyte subsets in predicting the response to biological therapeutics. Transmembrane tumor necrosis factor α (TNF-α)-bearing lymphocytes, CD4^+^ T cells, CD8^+^ T cells, and Plasma cells are found to have the potential to predict the response to anti-TNFα agents (16–18, 20–23), whereas memory T cells, CD4^+^ T cells, and naïve B cells may predict the response to vedolizumab (32–35). These findings provide evidence for both the mechanism of action of therapeutic agents and the clinical predictive potential of lymphocyte subsets. If validated in further high-quality studies, these findings could promote individual management of patients with IBD by providing clinicians with information regarding likely treatment efficacy before or at the start of treatment. Most recently, a randomized trial according to the application of biomarker-stratified interventional design in IBD (62) reported the failure to demonstrate the clinical utility of a CD8 T-cell transcriptional signature which was previously represented to be associated with the requirement for treatment escalation in IBD (63) but was challenged by later study (64). The plot twists underline the necessity to consider randomized trials as a mandatory step prior to the clinical implementation of prognostic biomarkers.

Compared to the prediction of particular therapies, it is more challenging but of greater clinical significance for the construction of a predictive tool to assess the risk–benefit ratios of several alternative biological therapeutics. A bioinformatics study validated the immune cell module score, GIMATS module mentioned above (8), in the prediction of various biologics therapies. Strikingly, the GIMATS module score succeeded in classifying patients based on the risk of non-response to not only IFX/ADA but also vedolizumab (19). Such prediction of therapeutic performance would have a significant impact on clinical decision-making. Therefore, the development of integrated predictive models is critical. Furthermore, with more biologics targeting various signaling pathways have been developed for the treatment of IBD, such as ustekinumab and Janus kinase inhibitors (65), the linkage between cellular heterogeneity and therapy resistance and the potential capability of lymphocyte subsets to predict the response to the newest agents is also warranted to be studied.

This study has some limitations. First, a considerable proportion of the studies were pilot cohorts with small sample size and strict enrollment criteria, especially in the field of predicting treatment response, which may affect the robustness and reliability of these findings. Also, we failed to implement meta-analyses because of the insufficient data from studies focusing on the same lymphocyte subsets. Therefore, it is essential to validate the results of these studies in large real-world populations prior to clinical application. The second major limitation was the lack of comparability between the prognosis or therapeutic response of groups with distinct baseline levels of lymphocyte subsets of interest, which was mainly due to the lack of adjustment for potential confounders. Third, the primary objective in a few studies was not limited to the predictive value of lymphocyte subpopulations. These studies mainly focused on other biomarkers, such as the products of lymphocytes and genes when discussing the predictive capacity. Hence did not implement statistical methods with more direct clinical significance to reveal the performance of certain lymphocyte subsets. Fourth, there is a lack of studies on the predictive role of lymphocyte subsets in the response of ustekinumab and small molecule drugs (such as JAK inhibitors). More research is needed to explore the capacity of lymphocyte subsets in predicting the therapeutic outcomes of these advanced treatment.

In conclusion, various lymphocyte subsets are associated with disease prognosis and therapeutic response in patients with IBD. Large-scale prospective studies and well-designed randomized trials are warranted to verify these findings and test the clinical utility before clinical application.

## Data Availability

The original contributions presented in the study are included in the article/**Supplementary Material**. Further inquiries can be directed to the corresponding authors.
