# Supplementary material for: Lymphocyte subsets for predicting inflammatory bowel disease progression and treatment response: a systematic review
Source: Front Immunol. 2024 Aug 20;15:1403420. doi: 10.3389/fimmu.2024.1403420 (PMC11368782; doi:10.3389/fimmu.2024.1403420)
Supplement: Supplementary file 1 [file Table1.docx]

**Supplementary material**

**Supplementary Table 1. Search strategy**

| **Search** | **Query** | **Items found** |
| --- | --- | --- |
| **PubMed Session Results (8 Apr 2023)** | | |
| #1 | (inflammatory bowel disease[MeSH Terms]) OR (crohn's disease[MeSH Terms]) OR (ulcerative colitis[MeSH Terms]) OR (crohn disease) OR (crohns disease) | 109,327 |
| #2 | (Lymphocytes[MeSH Terms]) OR (T cell OR T lymphocyte OR “Th1” OR “Th2” OR “Th17” OR “Treg” OR “Tfh” OR T follicular helper) OR (NK cell OR natural killer cell) OR (NKT cell) OR (B cell OR B lymphocyte OR plasma cell OR plasmacyte OR “Breg” OR B1 cell OR B1 lymphocyte OR B2 cell OR B2 lymphocyte) | 1,205,000 |
| #3 | prognosis OR relapse OR risk factor OR recurrence OR remission OR healing OR complication OR progression OR hospitalization OR surgery OR resection OR colectomy OR death OR mortality OR bowel damage OR exacerbation OR aggravation OR response OR responder | 17,023,360 |
| #4 | #1 AND #2 AND #3 | 5,745 |
| #5 | #4 NOT (((systematic review[Publication Type]) OR (review[Publication Type]) OR (comment[Publication Type]) OR (letter[Publication Type]) OR (editorial[Publication Type]) OR (case report[TI])) OR (((animals[MeSH]) OR (animal experimentation[MeSH])) NOT (humans[MeSH]))) AND (English[Language]) | 3,241 |
| **Embase (Ovid) Session Results (8 Apr 2023)** | | |
| #1 | 'inflammatory bowel disease'/exp OR 'inflammatory bowel disease' OR 'crohn disease'/exp OR 'crohn disease' OR 'ulcerative colitis'/exp OR 'ulcerative colitis' | 225,761 |
| #2 | 'lymphocyte'/exp OR 'lymphocyte' OR 't lymphocyte'/exp OR 't lymphocyte' OR 'th1 cell'/exp OR 'th1 cell' OR 'th2 cell'/exp OR 'th2 cell' OR 'th17 cell'/exp OR 'th17 cell' OR 'regulatory t lymphocyte'/exp OR 'regulatory t lymphocyte' OR 'tfh cell'/exp OR 'tfh cell' OR 'natural killer cell'/exp OR 'natural killer cell' OR 'natural killer t cell'/exp OR 'natural killer t cell' OR 'b lymphocyte'/exp OR 'b lymphocyte' OR 'plasma cell'/exp OR 'plasma cell' OR 'regulatory b lymphocyte'/exp OR 'regulatory b lymphocyte' OR 'b1 cell'/exp OR 'b1 cell' OR 'b2 cell'/exp OR 'b2 cell' | 1,295,153 |
| #3 | 'prognosis' OR 'relapse' OR 'risk factor' OR 'recurrence risk' OR 'recurrence free survival' OR 'remission' OR 'healing' OR 'complication' OR 'progression' OR 'hospitalization' OR 'surgery' OR 'resection' OR 'colectomy' OR 'death' OR 'mortality' OR 'bowel damage' OR 'exacerbation' OR 'aggravation' OR 'response' OR 'responder' | 1,295,153 |
| #4 | #1 AND #2 AND #3 | 13,309 |
| #5 | #4 AND english:la NOT (comment*:ti OR letter:it OR editorial:it OR 'conference abstract':it OR 'conference paper':it OR 'case report'/exp OR 'clinical trial (topic)'/exp OR 'clinical trial'/exp OR trial*:ti OR ((case NEXT/1 report*):ti) OR 'case series':ti OR ((case NEXT/1 histor*):ti) OR (case:ti AND next:ti AND stud*:ti)) AND ([humans]/lim OR [clinical study]/lim) | 5,481 |
| **Web of Science (Core Collection) Session Results (8 Apr 2023)** | | |
| #1 | TS = (“inflammatory bowel disease*” OR "IBD*" OR “Crohn disease” OR “Crohn's disease” OR “Crohns disease” OR “regional enteritis” OR “ulcerative colitis” OR “colitis ulcerosa”) | 154,873 |
| #2 | TS = (lymphocyte OR (“T cell*” OR “T lymphocyte*” OR “Th1 cell*” OR “Th2 cell*” OR “Th17 cell*” OR “Treg cell*” OR “Tfh cell*” OR “T follicular helper cell*”) OR (“NK cell*” OR “natural killer cell*”) OR (“NKT cell*”) OR (“B cell*” OR “B lymphocyte*” OR “plasma cell*” OR plasmacyte OR “Breg cell*” OR “B1 cell*” OR “B1 lymphocyte*” OR “B2 cell*” OR “B2 lymphocyte*”)) | 812,018 |
| #3 | TS = (prognosis OR relapse OR "risk factor*" OR recurrence OR remission OR healing OR complication OR progression OR hospitalization OR surgery OR resection OR colectomy OR death OR mortality OR "bowel damage" OR exacerbation OR aggravation OR response OR responder) | 9,061,058 |
| #4 | #1 AND #2 AND #3 | 8,535 |
| #5 | ((#4) NOT DT = (Editorial Material OR Letter OR Meeting Abstract OR Meeting Summary OR Review)) AND LA=(English) | 6,143 |

**Supplementary Table 2. Study quality assessment by the Newcastle-Ottawa score**

| Study | Selection | | | | Comparability | Outcome | | | Score |
| --- | --- | --- | --- | --- | --- | --- | --- | --- | --- |
|  | Representativeness of the exposed cohort | Selection of the non-exposed cohort | Ascertainment of exposure | Demonstration that outcome of interest was not present at start of study | Comparability of cohorts on the basis of the design or analysis | Assessment of outcome | Was follow-up long enough for outcomes to occur | Adequacy of follow up of cohorts |  |
| Allez et al.^30^ | 1 | 1 | 1 | 1 | 0 | 1 | 1 | 1 | 7 |
| Amini et al.^23^ | 1 | 1 | 1 | 1 | 0 | 1 | 1 | 1 | 7 |
| Andreu-Ballester et al.^26^ | 1 | 1 | 1 | 0 | 0 | 1 | 0 | 1 | 5 |
| Boschetti et al.^25^ | 1 | 1 | 1 | 1 | 0 | 1 | 1 | 1 | 7 |
| Chao et al.^27^ | 1 | 1 | 1 | 1 | 0 | 1 | 1 | 1 | 7 |
| Coletta et al.^34^ | 1 | 1 | 1 | 1 | 2 | 1 | 1 | 1 | 9 |
| Dai et al.^24^ | 0 | 1 | 1 | 0 | 0 | 0 | 0 | 1 | 3 |
| Di Sabatino et al.^20^ | 1 | 1 | 1 | 1 | 0 | 1 | 1 | 1 | 7 |
| Dige et al.^16^ | 1 | 1 | 1 | 1 | 0 | 1 | 0 | 1 | 6 |
| Duclaux-Loras et al.^29^ | 1 | 1 | 1 | 1 | 2 | 1 | 1 | 1 | 9 |
| Dulic et al.^22^ | 1 | 1 | 1 | 1 | 0 | 1 | 1 | 1 | 7 |
| Gaujoux et al.^18^ | 1 | 1 | 1 | 1 | 1 | 1 | 1 | 1 | 8 |
| Gonzalez-Vivo et al.^35^ | 1 | 1 | 1 | 1 | 0 | 1 | 1 | 1 | 7 |
| Kotsafti et al.^31^ | 1 | 1 | 1 | 0 | 2 | 1 | 0 | 0 | 6 |
| Li et al.^21^ | 1 | 1 | 1 | 1 | 0 | 1 | 1 | 1 | 7 |
| Magnusson et al.^17^ | 1 | 1 | 1 | 1 | 0 | 1 | 1 | 1 | 7 |
| Shi et al.^19^ | 1 | 1 | 1 | 1 | 0 | 1 | 1 | 1 | 7 |
| Smids et al.^28^ | 1 | 1 | 1 | 1 | 0 | 1 | 1 | 1 | 7 |
| Ungar et al.^32^ | 1 | 1 | 1 | 1 | 0 | 1 | 1 | 0 | 6 |
| Verstockt et al.^33^ | 1 | 1 | 1 | 1 | 0 | 1 | 1 | 0 | 6 |
